# Association Between Rapid Progression, Early Mortality, and Imaging in Neonatal-Onset Alexander Disease

**DOI:** 10.1055/a-2773-9382

**Published:** 2025-12-24

**Authors:** Simone Schwarz, Sylke J. Steggerda, Linda S. de Vries, Katharina Schulz, Nikola R. Dürr, Maarten H. Lequin, Thorsten Rosenbaum, Ursula Felderhoff-Müser, Robin-Tobias Jauss, Francisco Brevis Nuñes, Nora Bruns

**Affiliations:** 1 Department of Neonatology and Pediatric Intensive Care Medicine, Sana Clinics Duisburg, Duisburg, Germany; 2 Department of Pediatrics I, University Hospital Essen, University of Duisburg-Essen, Essen, Germany; 3 TNBS, Centre for Translational Neuro- and Behavioral Sciences, University Hospital Essen, University of Duisburg-Essen, Essen, Germany; 4 Department of Pediatrics, Division of Neonatology, Willem-Alexander Children's Hospital, Leiden University Medical Center, Leiden, the Netherlands; 5 Clinic for Radiology and Neuroradiology, Sana Clinics Duisburg, Duisburg, Germany; 6 Department of Radiology, Edward B Singleton, Texas Children's Hospital, Austin, Texas, United States; 7 Department of Pediatrics, Sana Clinics Duisburg, Duisburg, Germany; 8 Institute of Human Genetics, University of Leipzig Medical Center, Leipzig, Germany

**Keywords:** neonatal leukodystrophy, neonatal onset, Alexander disease, CUS, white matter disease, neonatal seizures

## Abstract

Alexander disease represents a rare genetic leukodystrophy caused by abnormal astrocytic accumulations of intracytoplasmic proteinaceous inclusions with astrocyte dysfunction. With neonatal onset, survival ranges from 1.5 months to more than 7.5 years, with a possible association between the underlying point mutation, the level of protein accumulation in the cerebral white matter, disease progression, and survival time. We describe the clinical and cerebral imaging features of a female newborn with neonatal-onset Alexander disease caused by a heterozygous de novo point mutation c.1106T > C; p.(Leu369Pro) located in the coil 2B area of the glial fibrillary acidic protein (GFAP). Early-onset seizures, lethargy, and rapid loss of spontaneous movements were accompanied by rapidly evolving brain morphologic abnormalities and early death. The progression of cerebral abnormalities was monitored by magnetic resonance imaging and serial cranial ultrasound exams. As shown in this case study and the accompanying literature review, rapid accumulation of GFAP, as indicated by volume expansion of affected structures on brain imaging, combined with early onset of seizures and rapid clinical deterioration, seems to be associated with poor prognosis. In this case, high-resolution ultrasound offered an easily accessible, serial bedside imaging tool for the detection and follow-up of pathognomonic features of Alexander disease. We observed a close genotype-phenotype interaction in neonatal-onset Alexander disease, with the c.1106T > C; p.(Leu369Pro) mutation in coil2B associated with early death.

## Introduction

Alexander Disease (OMIM #203450, ALXDRD) is a very rare genetic leukodystrophy caused by a dominant gain-of-function mutation in the glial fibrillary acidic protein (GFAP) gene on chromosome 17q21. 1 GFAP, as the major intermediate filament of astrocytes, is involved in the morphology and motility of astrocytes and in the interaction between astrocytes and oligodendrocytes. 2 Overexpression and reduced degeneration of GFAP, along with upregulation of heat shock proteins, cause accumulation of filaments in the cytoplasm of astrocytes, called “Rosenthal fibers”, leading to astrocyte dysfunction. 3 4 The spectrum of clinical manifestations of ALXDRD and the severity of the disease are closely related to the age of onset. 5 Cases with neonatal manifestation are characterized by rapid onset and progression within the first month of life, leading to severe disability and death mostly within 2 years. Frequent and intractable seizures occur as an early and obligatory symptom in combination with severe motor and cognitive impairment. Obstructive hydrocephalus due to aqueductal stenosis is caused by a typical progressive volume increase of periaqueductal white matter accompanied by increased cerebrospinal fluid protein content. 6 In the neonatal period, disease progression manifests as progressive muscle hypotonia and loss of the ability to suck. 7 Cranial magnetic resonance (MR) imaging shows typical white matter abnormalities with frontal predominance, extensive periventricular enhancement, and involvement of the basal ganglia and cerebellum. To date, 21 cases of neonatal-onset ALXDRD have been described, with survival ranging from 1.5 months to more than 7.5 years, making parental counseling complex. 2 3 8 9 Some studies have suggested an association between genotype and disease severity, which may partially explain the variation in survival times. 5 9 ALXDRD, like other genetic white matter disorders, has pathognomonic MR imaging features that allow noninvasive diagnosis based on pattern recognition. 10 11 12 Even in the era of readily available genetic testing, such as whole-exome sequencing, pattern recognition continues to play an important role in narrowing down possible diagnoses. The relevance of neuroimaging and the potential role of bedside ultrasound for diagnosis and prognostic prediction in neonatal onset ALXDRD are evaluated in this case study. 13

## Methods

### Aims, Design, and Study Setting

This retrospective case study conducted at Sana Hospital Duisburg aims to elaborate on the prognostic interplay of clinical parameters, cerebral imaging progression, and the underlying mutation for outcome prediction in neonatal onset ALXDRD. In addition, the potential of sonographic imaging features for diagnosis and follow-up will be assessed. We collected clinical, genetic, and longitudinal imaging findings, compared these data with other published cases, and drew conclusions for outcome prediction.

### Cerebral Imaging

Cranial ultrasound (CUS) was performed serially using a high-end ultrasound device (GE Logiq E10s R3, GE Healthcare, Boston, Massachusetts, United States) equipped with high-resolution mini-curved and linear transducers (GE C3-10-D, GE ML4-20-D), according to recommended standards, 14 15 with the highest quality of the examination being obtained on day of life (DOL) 11 and 32. These images were retrospectively analyzed by two independent experts (LSDV and SJS) using the ALXDRD MRI diagnostic criteria according to van der Knaap et al. 11 12

MRI was performed on DOL 4 and 12, including T1- and T2-weighted images with and without gadolinium contrast, diffusion-weighted images, and susceptibility-weighted images. These images were also retrospectively analyzed by experienced neuroradiologists (KS and NRD), blinded to the CUS studies, using the same ALXDRD MRI diagnostic criteria by van der Knaap.

Because the comparison of CUS on DOL 11 and MRI on DOL 12 yielded consistent results, further imaging progression was monitored with CUS.

### Statistical Analysis

Cohen's Kappa 16 was calculated to determine the agreement between MRI and CUS findings using Python 3.13 (Python Software Foundation, Beaverton, Oregon, United States) in a Jupyter Lab environment (Version 4.3.4; Project Jupyter). 17 Ratings categorized as “not applicable” were excluded from the analyses. This was determined when the corresponding structures could not be evaluated due to image quality or were not visible in the stored images.

## Results

### Clinical Report

The girl was born at 39 ^1/7^ weeks' gestation by uncomplicated vaginal delivery as the third child of nonconsanguineous parents (weight 3.59 kg [50–75 percentile], height 53 cm [75–90 percentile], head circumference 35 cm [25–50 percentile]). There were no abnormal findings on prenatal ultrasound, and pregnancy and family history were unremarkable. On her first DOL, she presented with muscular hypotonia and respiratory failure requiring admission to the local neonatal intensive care unit (NICU). Seizures began on DOL 2. On DOL 8, the girl was transferred to our NICU with suspected congenital infection. Symptoms progressed rapidly with loss of spontaneous movements and inability to suck. She became lethargic, hypothermic, and bradypnoeic until she died at 8 weeks of age. During these 8 weeks, a continuous increase in the head circumference caused by an obstructive hydrocephalus was observed. Cerebrospinal fluid protein was elevated, but in-depth laboratory tests were unremarkable. The rapid clinical deterioration and morphologic imaging findings mimicked an inflammatory process, suggesting congenital infection as a potential and common cause. Metabolic and genetic diseases with leukoencephalopathy, especially mitochondrial diseases such as Leigh syndrome, were also considered.

### Cranial Ultrasound

**Fig. 1:**
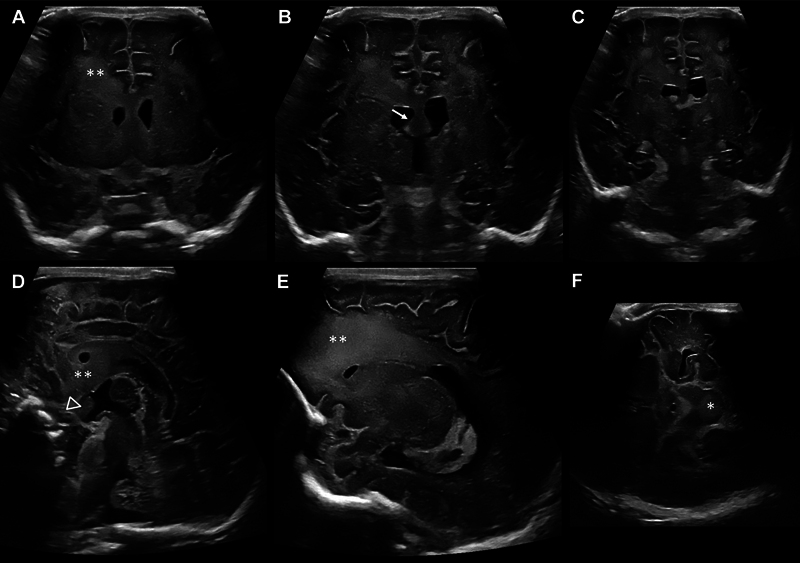
Cranial ultrasound on DOL 11. Increase of white matter echogenicity with very fine, homogeneous echo texture (two asterisks) with frontal predominance ( **A–E** ), volume expansion of the fornix area (arrow) ( **B** ), the area of the optic chiasma between optic recess and infundubulum of the third ventricle (arrow head) ( **D** ), and midbrain involvement (asterisk) ( **F** ) in coronar ( **A–C** ), sagittal ( **D, E** ), and axial ( **F** ) planes.

**Fig. 2:**
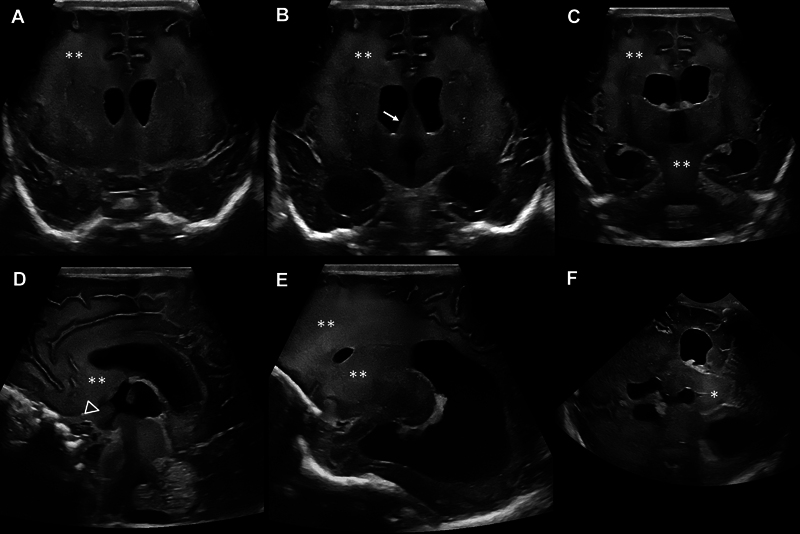
Cranial ultrasound on DOL 32. Progressive increase of white matter echogenicity with very fine, homogeneous echo texture (two asterisks) with frontal predominance ( **A–E** ), volume expansion of the fornix area (arrow) ( **B** ), the area of the optic chiasma between optic recess and infundubulum of the third ventricle (arrow head) ( **D** ), and midbrain involvement (asterisk) ( **F** ) with obstructive hydrocephalus ( **A–F** ) in coronar ( **A–C** ), sagittal ( **D, E** ), and axial ( **F** ) planes.

**Fig. 3:**
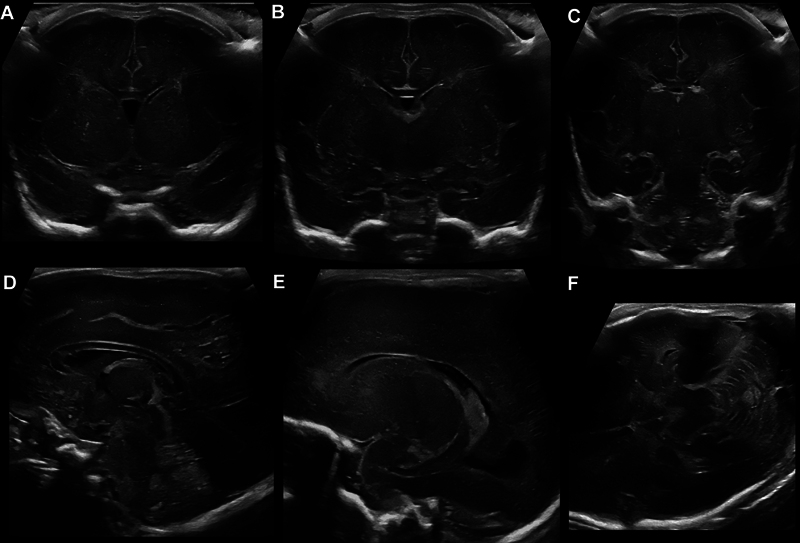
Normal cranial ultrasound images. Normal cranial ultrasound images of a former preterm infant at 35 weeks of gestation in coronal ( **A–C** ), sagittal ( **D, E** ), and axial ( **F** ) planes for comparison.

The first abnormality on CUS was a symmetrical increase of white matter echogenicity with frontal predominance and transition to the anterior corpus callosum and septum pellucidum/septal nuclei extending to striatum and basal forebrain structures ( Fig. 1A–E ). More detailed examinations revealed midbrain involvement ( FIG. 1F ). Rapid progression of imaging abnormalities was observed on serial CUS with increasing echogenicity and volume increase/swelling of the aforementioned structures, including the midbrain with occlusion of the aqueduct and development of internal hydrocephalus ( Fig. 2 ). Fig. 3 shows normal CUS images for comparison purposes.

### MR Imaging

**Fig. 4:**
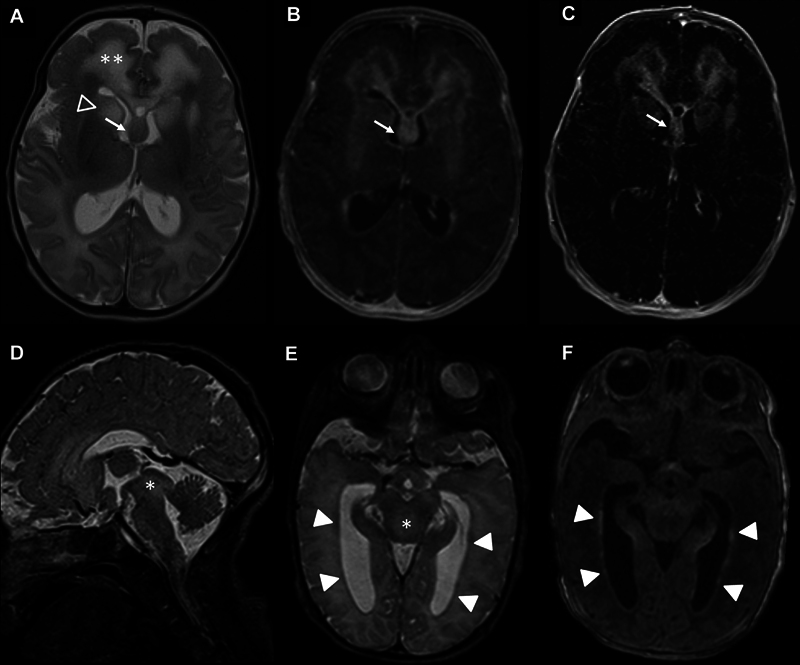
MR imaging on DOL 12. ( **A** ) T2W image with white matter abnormalities with frontal predominance (two asterisks), involvement of the head of the caudate nucleus (open arrow head), and thickened fornix (arrow). ( **B** ) Contrast enhancement on the T1W image with enhancement of the frontal white matter, head of the caudate nucleus, and fornix. ( **C** ) Contrast enhancement on the T1W subtraction image with enhancement of the frontal white matter, head of caudate nucleus, and fornix, and cerebral white matter spots. ( **D** ) Involvement of the midbrain with volume expansion (asterisk) on sagittal T2W image. ( **E** ) Periventricular rim of low signal (arrow heads) and involvement of the midbrain (asterisk) on T2W image. ( **F** ) Periventricular rim of high signal on T1W image (arrow heads).

MR imaging showed typical signs of ALXDRD with 5/5 criteria fulfilled + 1/10 additional criterion for the diagnosis of ALXDRD on DOL 4 with rapid progression to 5/5 criteria + 4/10 additional criteria on DOL 12 ( Fig. 4 ; Table 1 ).

**Table 1:** Criteria for ALXDRD in MRI and CUS

Criterion	MRI DOL 4	MRI DOL 12	CUS DOL 11	CUS DOL 32
Criterion 1: WM abnormalities and frontal preponderance	+	++	++	+++
WM abnormalities	+	++	++	+++
Frontal predominance	+	++	++	++
Involvement of periventr. WM	+	++	++	++
Involvement of deep WM	+	++	++	+++
Involvement of subcortical WM	−	−	−	+
Swelling of the abnormal WM	−	+	+	++
Atrophy of the abnormal WM	−	−	−	−
Cystic degeneration of the abnormal WM	+	+	+	−
Criterion 2: Periventricular rim	+	++	(+)	−
Periventricular rim of low signal on T2 and high signal on T1	+	++	(+)	−
			Rim of low echogenicity	
Criterion 3: basal ganglia and thalami	+	++	++	++
Involvement of central nuclei	+	++	++	++
Head of the caudate nucleus	+	++	++	++
Putamen	+	+	+	+
Globus pallidus	+	+	+	+
Thalamus	−	−	+	+
Aspect of central nuclei	Abnormal	Abnormal	Abnormal	Abnormal
Elevated signal on T2W images	−	−	NA	NA
Elevated signal on T1W images	+	+	NA	NA
Swelling	+	++	++	++
Atrophy	−	−	−	+
Criterion 4: brainstem lesions	+	++	++	+++
Brainstem lesions	+	++	++	+++
Midbrain	+	++	++	+++
Pons	−	−	−	+
Medulla	−	+	−	−
Nodular lesions with mass effect	−	−	−	−
Brainstem atrophy	−	−	−	−
Criterion 5: contrast enhancement	+	++	NA	NA
Contrast enhancement	+	++	NA	NA
Cerebral WM spots	+	++	NA	NA
Ependymal lining	+	+	NA	NA
Brainstem lesions	+	++	NA	NA
Lesions middle cerebellar peduncles	+	−	NA	NA
Dentate nucleus	NA	−	NA	NA
Chiasm	+	++	NA	NA
Fornix	+	++	NA	NA
Extra features				
Enlargement of the lateral ventricles	−	+	+	++
Involvement of cerebellar structures	−	−	−	(+)
				Flattened hemispheres
Cerebellar hemispheric WM abnormalities	−	−	NA	NA
Hilus of the dentate nucleus abnormalities	+	+	NA	NA
Dentate nucleus	−	−	NA	NA
Middle cerebellar peduncles	−	−	NA	NA
Cerebellar swelling	−	−	−	−
Cerebellar atrophy	−	−	−	+
Thickened fornix	NA	+	(+)	(++)
			Thickened septal grey nuclei/Septum pellucidum/fornix area	
Thickened chiasm	NA	+	(+)	(++)
			Space-occupying lesion in the area of the optic chiasma between the optic recess and infundubulum of the third ventricle	

Abbreviations: CUS, cranial ultrasound; DOL, day of life; MRI, magnetic resonance imaging; NA, not applicable; WM, white matter.

### Retrospective Comparison of Cranial Ultrasound and MR Imaging

Evaluation of CUS imaging revealed typical signs of ALXDRD with 4/5 criteria and 3/10 additional criteria for the diagnosis of ALXDRD fulfilled on DOL 11 and 3/5 criteria and 5/10 additional criteria on DOL 32, with rapid progression of the extent of most abnormalities ( Table 1 ). The agreement between MRI and ultrasound was almost perfect, with Cohen's Kappa = 0.93 between the examinations on DOL 11/12, with less than 24 hours' time difference between the two examinations. CUS was inferior to MR imaging for detailed assessment of brainstem and posterior fossa structures, resulting in inapplicable criteria for some imaging features ( Table 1 ). In addition, no comparison could be made with MRI findings using gadolinium as a contrast agent because there is currently no comparable application for ultrasound. However, ultrasound revealed additional abnormalities in follow-up examinations that may also be helpful to characterize the pattern of affected cerebral structures ( Figs. 1 and 2 ; Table 2 ).

**Table 2:** Additional findings on cerebral ultrasound compared to MRI

CUS findings	CUS DOL 11	CUS DOL 32
Very fine, homogeneous echo texture of affected structures with increased echogenicity	+	++
Volume expansion of affected structures, including the midbrain, with compression of the aqueduct	+	++

Abbreviations: CUS, cranial ultrasound; DOL, day of life.

### Genetic Testing

A heterozygous, pathogenic de novo point mutation c.1106T > C; p.(Leu369Pro) located in the coil 2B area of GFAP was identified via trio whole exome sequencing from peripheral blood samples of the infant and her parents. This variant has been previously described in one patient with neonatal-onset ALXDRD and early death ( Table 3 ). 9 No other clinically relevant variant could be identified, including analysis of mitochondrial DNA.

**Table 3:** Neonatal-onset ALXDRD caused by a mutation in the coil 2B area of GFAP

	Case study	Knuutinen et al 9	Paprocka et al 2	Mura et al 8	Takeuchi et al 3	Li et al 4			
						Pat. 32	Pat. 33	Pat. 35	Pat. 36
cDNA mutation	c.1106T > C	c.1106T > C	c.1187C > T	c.1187C > T	c.1097A > G	c.1049_1050insCTTGCA	c.1055T > C	c.1090G > C	c.1096T > C
Protein mutation	p.(Leu369Pro)	p.(Leu369Pro)	p.(Thr396Ile)	p.(Thr396Ile)	p.(Tyr366Cys)	p.(Tyr349_Gln350insHisLeu)	p.(Leu352Pro)	p.(Ala364Pro)	p.(Tyr366His)
Protein structure	Coil2B	Coil2B	Coil2B	Coil2B	Coil2B	Coil2B	Coil2B	Coil2B	Coil2B
Age of onset	First DOL	4th DOL	First week	First month	Ventriculomegaly at 36 wk of gestation	Onset at 1 mo of age	First DOL	First month	First month
First signs	Muscular hypotonia, respiratory failure	Drug-resistant seizures	Increasing apathy, severely limited spontaneous activity, and a lack of eye tracking	NA	Hydrocephalus	Severe vomiting, intractable seizures	Feeding problems	NA	NA
Seizures	Second DOL, drug resistent	Fourth DOL, drug resistent	Drug-resistant seizures	NA	6 mo	Drug resistant	Yes	Yes	Yes
Progression	Rapid progression with seizures, adynamie, loss of spontaneous movements, and inability to suck, macrocrania with hydrocephalus	Rapid progression with lethargy and prolonged seizures, macrocrania with hydrocephalus	Rapid progression, drug-resistant seizures, makrocrania with hydrocephalus	Very severe, rapidly evolving course with absence of postural acquisition, hydrocephalus	Psychomotor development significantly impaired, no neck control, no vocalization, lack of eye tracking, macrocrania with hydrocephalus	Devastating course, macrocephaly	Rapid progression	Macrocephaly	NA
First MRI findings	See Table 1	WM loss and hydrocephalus, caused by aqueductal stenosis due to enlargement of the tectum, extensive signal abnormalities	Decreased T1 and increased T2 WM signals in both hemispheres with frontal domination, midbrain, cortico-spinal tracts, and enlarged basal ganglia with heterogeneous signals and enhancement, DWI with generalized abnormality and signal with increased diffusivity in the frontal white matter	Typical MRI features with prominent cerebral WM abnormalities with antero-posterior gradient and basal ganglia involvement	Narrow aqueductus cerebri, ventriculomegaly, abnormal WM signal intensity	Typical	Typical	NA	Typical
Follow-up MRI	See Table 1	NA	1-wk follow-up generalized WM T2 hyperintensity with a low signal rim around ventricles, marked symmetrical perivascular spaces in internal capsules 4-mo follow-up progression of abnormal, diffused, increased signal of WM with frontal domination, periventricular narrow bands of high T1 and low T2 signals, inhomogeneous signals from the basal nuclei with the most intense changes in the heads of the caudate nuclei, lenticular nuclei, and anteromedial parts of the thalamus, postcontrast enhancement of subcortical nuclei and along the cortico-spinal tracts	Hydrocephalus, early and rapid cystic degeneration	5-mo follow-up hypomyelination and periventricular cavitation localized to the frontal area	NA	NA	NA	NA
CUS findings	See Table 1	Hydrocephalus, increasing WM abnormalities	Enlarged supratentorial ventricular system	NA	NA	NA	NA	NA	NA
Time point of death	8 wk	6 wk	4 mo	Within the second year	Alive with 1 y of life	3.5 mo	38 d	4 mo	With 1.5 y

Abbreviations: CUS, cranial ultrasound; DOL, day of life; MRI, magnetic resonance imaging; NA, not applicable; WM, white matter.

## Discussion

This case study highlights the interplay between clinical presentation, imaging features, and genetic testing for diagnosis and prognostication in rare neonatal diseases like genetic leukodystrophies. We present the first direct comparison between high-resolution CUS, the first-line imaging modality in neonates, and MR imaging, the established imaging method for diagnosing ALXDRD, in neonatal onset ALXDRD. Showing an almost perfect agreement between pattern recognition in MR imaging and ultrasound using the ALXDRD criteria described by van der Knaap et al. 12 this case may help in pattern recognition using bedside CUS.

Diagnosis of neonatal ALXDRD can be made based on clinical symptoms and typical brain imaging findings, and subsequently confirmed by genetics. Prior to the availability of genetic diagnosis, MRI criteria established by van der Knaap et al. allowed the diagnosis of ALXDRD without cranial biopsy. 12 The diagnosis is confirmed when four out of five main criteria are present, with very limited data in neonatal onset ALXDRD. In this case, a pediatric neuroradiologist aware of these criteria suggested the diagnosis, which was confirmed by whole exome sequencing.

Cerebral pathologies in ALXDRD are mainly caused by the accumulation of intracytoplasmic proteinaceous inclusions in astrocytes with varying degrees in different brain regions, causing pathologies first in areas with high GFAP content. 18 In neonatal onset ALXDRD, hydrocephalus has been reported in association with a markedly reduced aqueduct. This occurs in the context of diffuse astrocytosis, with reactive astrocytes displaying abundant cytoplasm containing Rosenthal fibers, particularly in a subependymal distribution, which may predispose to aqueductal narrowing. 19 In this case, we observed a volume increase of affected structures in serial imaging. The increase in periaqueductal white matter volume detected on DOL 32 was associated with increased echogenicity on CUS with a very fine, homogeneous echo texture, similar to the previously observed frontal pathologies. Other affected structures exhibited progressive volume expansion with increasingly irregular morphology of the lateral and third ventricles, suggesting swelling due to toxic destruction or rapid accumulation of pathological intracytoplasmic proteins that correlated with rapid clinical deterioration.

Several studies have reported a strong genotype-phenotype association in ALXDRD. 5 9 Especially in neonatal onset ALXDRD mutations in the coil 2B domain seem to be linked to early death. To date, only one other case with the same point mutation c.1106T > C; p.(Leu369Pro) in coil2B of the GFAP gene has been described. 9 The clinical course was identical, with progressive white matter lesions on cranial imaging, occlusive hydrocephalus due to aqueductal stenosis, rapid clinical deterioration, and death at 6 weeks of age.

Presumably, certain mutations like the point mutation c.1106T > C;p.(Leu369Pro) lead to specific gain-of-function mutations that confer qualitatively increased toxicity or rapid accumulation of GFAP in astrocytes and thus to neonatal presentation. Animal studies with overexpression models demonstrate a toxic threshold, where mice with the highest GFAP expression died at 3 to 5 weeks due to early seizures characteristic of neonatal onset ALXDRD. 18 The increase in echogenicity and the expansion of the affected areas on CUS, accompanied by clinical deterioration, suggest that brain injuries induced by GFAP could possibly be monitored using high-resolution ultrasound.

Modern high-resolution, high-end ultrasound equipment allows improved visualization of intracranial structures with high spatial and temporal resolution. In this study, the progression of morphological changes associated with neonatal onset ALXDRD was monitored via point-of-care ultrasound and showed excellent intermodal agreement with MR imaging. Point-of-care CUS can be performed serially, is broadly available around the clock, and does not require transport and/or sedation. As a relevant limitation, the brain stem and the posterior fossa can only be partially visualized, and there is no equivalent to a gadolinium contrast agent. Nonetheless, high-resolution ultrasound may be a cost-effective noninvasive bedside alternative to MRI for diagnosis and serial monitoring of white matter abnormalities. In low-income countries, imaging may be a target for even lower-cost genetic testing.

In neonatal-onset ALXDRD, integrating multimodal data from clinical assessments, imaging studies, and genetic analyses enables monitoring of disease progression, facilitating prognostication of life expectancy, which in turn informs treatment planning and parental counseling. Early prognostication could enable timely palliative care discussions with parents, avoid unnecessary invasive diagnostics and interventions, and facilitate home discharge, allowing the family to spend quality time together.

## Conclusion

High-resolution bedside ultrasound should be included in the diagnostic workup for neonatal leukodystrophies, such as ALXDRD, to recognize patterns and facilitate follow-up. The c.1106T > C;p.(Leu369Pro) point mutation seems to be associated with a very poor prognosis and early death, which underscores the importance of genotype-phenotype correlation in neonatal-onset ALXDRD.

## References

[1] Vázquez E Macaya A Mayolas N Arévalo S Poca M A Enríquez G Neonatal Alexander disease: MR imaging prenatal diagnosis AJNR Am J Neuroradiol 2008 29 10 1973 1975 18653683 10.3174/ajnr.A1215 PMC8118950

[2] Paprocka J Nowak M Machnikowska-Sokołowska M Rutkowska K Płoski R Leukodystrophy with macrocephaly, refractory epilepsy, and severe hyponatremia-the neonatal type of Alexander disease Genes (Basel) 2024 15 03 350 38540409 10.3390/genes15030350 PMC10970303

[3] Takeuchi H Higurashi N Kawame H GFAP variant p. Tyr366Cys demonstrated widespread brain cavitation in neonatal Alexander disease Radiol Case Rep 2021 17 03 771 774 35003479 10.1016/j.radcr.2021.11.066 PMC8717161

[4] Li R Johnson A B Salomons G Glial fibrillary acidic protein mutations in infantile, juvenile, and adult forms of Alexander disease Ann Neurol 2005 57 03 310 326 15732097 10.1002/ana.20406

[5] Prust M Wang J Morizono H GFAP mutations, age at onset, and clinical subtypes in Alexander disease Neurology 2011 77 13 1287 1294 21917775 10.1212/WNL.0b013e3182309f72 PMC3179649

[6] Springer S Erlewein R Naegele T Alexander disease–classification revisited and isolation of a neonatal form Neuropediatrics 2000 31 02 86 92 10832583 10.1055/s-2000-7479

[7] Singh N Bixby C Etienne D Tubbs R S Loukas M Alexander's disease: reassessment of a neonatal form Childs Nerv Syst 2012 28 12 2029 2031 22890470 10.1007/s00381-012-1868-8

[8] Mura E Nicita F Masnada S Alexander disease evolution over time: data from an Italian cohort of pediatric-onset patients Mol Genet Metab 2021 134 04 353 358 34865968 10.1016/j.ymgme.2021.11.009

[9] Knuutinen O Kousi M Suo-Palosaari M Neonatal Alexander disease: novel GFAP mutation and comparison to previously published cases Neuropediatrics 2018 49 04 256 261 29801191 10.1055/s-0038-1649500

[10] Oikarainen J H Knuutinen O A Kangas S M Brain MRI findings in paediatric genetic disorders associated with white matter abnormalitiesDev Med Child Neurol2024 10.1111/dmcn.16036 PMC11695792 39080972

[11] van der Knaap M S Salomons G S Li R Unusual variants of Alexander's disease Ann Neurol 2005 57 03 327 338 15732098 10.1002/ana.20381

[12] van der Knaap M S Naidu S Breiter S N Alexander disease: diagnosis with MR imaging AJNR Am J Neuroradiol 2001 22 03 541 552 11237983 PMC7976831

[13] Schwarz S Steggerda S J de Vries L S Rapid progression and early mortality in neonatal-onset Alexander disease: association between clinical deterioration, cranial ultrasound and magnetic resonance imaging 10.21203/rs.3.rs-6572580/v12025 PMC12956381 41443241

[14] eurUS.brain group Dudink J Jeanne Steggerda S Horsch S State-of-the-art neonatal cerebral ultrasound: technique and reporting Pediatr Res 2020 87 01 3 12 10.1038/s41390-020-0776-y PMC7098885 32218539

[15] Maller V V Cohen H L Neonatal head ultrasound: a review and update-part 1: techniques and evaluation of the premature neonate Ultrasound Q 2019 35 03 202 211 30855418 10.1097/RUQ.0000000000000439

[16] Cohen J A coefficient of agreement for nominal scales Educ Psychol Meas 1960 20 01 37 46

[17] Kluyver T Ragan-Kelley B Pérez F, et al , Eds.Jupyter Notebooks - a publishing format for reproducible computational workflowsInternational Conference on Electronic Publishing;2016

[18] Messing A Brenner M Feany M B Nedergaard M Goldman J E Alexander disease J Neurosci 2012 32 15 5017 5023 22496548 10.1523/JNEUROSCI.5384-11.2012 PMC3336214

[19] Townsend J J Wilson J F Harris T Coulter D Fife R Alexander's disease Acta Neuropathol 1985 67(1–2):163 166 4040694 10.1007/BF00688138

